# Decreased Plasma Concentration of Hydrogen Sulfide in Hospitalized COVID-19 Patients: A Novel Determinant of Mortality?

**DOI:** 10.3390/antiox15030307

**Published:** 2026-02-28

**Authors:** Chiara Stranieri, Edoardo Giuseppe Di Leo, Elisa Danese, Roberta Poffe, Arianna Barbieri, Laura Pighi, Antonio Randon, Luciano Cominacini, Anna Maria Fratta Pasini

**Affiliations:** 1 Section of Internal Medicine D, Department of Medicine, University of Verona, P. le L.A. Scuro, 37134 Verona, Italy; chiara.stranieri@univr.it (C.S.); edoardogiuseppe.dileo@univr.it (E.G.D.L.); arianna.barbieri@studenti.univr.it (A.B.); antonio.randon@studenti.univr.it (A.R.); luciano.cominacini@univr.it (L.C.); 2 Section of Clinical Biochemistry, Department of Engineering for Innovation Medicine, University of Verona, 37134 Verona, Italy; elisa.danese@univr.it (E.D.); roberta.poffe@aovr.veneto.it (R.P.); laura.pighi@univr.it (L.P.)

**Keywords:** COVID-19, glutathione, hydrogen sulfide, N-acetylcysteine, oxidative stress

## Abstract

In this study, we first focused on measuring H_2_S and oxidative stress as indicators of in-hospital mortality observed within 24 h from admission in hospitalized non-survivor and survivor patients affected by COVID-19. Then, we analyzed whether N-acetylcysteine (NAC) can increase H_2_S and GSH concentrations in different cell lines. H_2_S levels were significantly increased in all COVID-19 patients (both survivors and non-survivors) compared to non-COVID-19 subjects (*p* = 0.0016), but non-survivors showed significantly lower H_2_S plasma levels than survivors (*p* = 0.008). Oxidative stress measured as circulating malondialdehyde (MDA) resulted in lower levels in non-COVID-19 subjects than in the two COVID-19 patient groups (*p* = 0.03). However, non-survivors had significantly higher plasma MDA than survivors (*p* = 0.0001). A Kaplan–Meier curve for H_2_S indicates a markedly reduced survival probability in COVID-19 patients with lower H_2_S levels (log-rank *p* = 0.004). NAC activity significantly reduced reactive oxygen species and lipid peroxidation induced by tert-butyl hydroperoxide in cultured cells (*p* from <0.01 to <0.001). Furthermore, NAC increased the cellular production of H_2_S (*p* < 0.01) and GSH (*p* < 0.01). These findings indicate the important prognostic role of H_2_S in COVID-19 patients at hospital admission and that NAC might be helpful in all clinical situations characterized by low levels of H_2_S.

## 1. Introduction

Coronavirus disease 2019 (COVID-19), caused by severe acute respiratory syndrome coronavirus-2 (SARS-CoV-2), turned out to be one of the major worldwide health emergencies of the 21st century [1]. Pandemic onset resulted in the demand for an effective and reliable design for treating infected patients, alleviating symptoms, and preventing critical disease. Because of their reported anti-inflammatory, antioxidant, and antiviral effects, reactive sulfur compounds, including hydrogen sulfide (H_2_S), and some other lesser-known sulfur compounds [2] have gained the attention of scientists for the treatment and prevention of the adverse effects of diseases caused by SARS-CoV-2.

H_2_S belongs to the class of labile biological mediators [2]. They share many similarities, such as fast transfer across cell membranes without specific transporters [3]. H_2_S is a pivotal signaling compound contributing to many pathological and physiological reactions [2]. H_2_S is synthesized primarily via cystathionine-beta-synthase (CBS), cystathionine-gamma-lyase (CSE/CTH), and cysteine-aminotransferase (CAT)-3-mercaptopyruvate sulfurtransferase (3-MST) [3,4,5,6]. These enzymes are known to metabolize methionine and cysteine to form H_2_S [2]. Eventually, generation of H_2_S can also take place nonenzymatically through reductive chemistry of different sulfur species [7] or catalysis of cysteine by synchronized activity of iron (Fe^3+^) and pyridoxal 5′-phosphate [8]. H_2_S has been reported to target four key points of SARS-CoV-2 infection [9]: (1) cell entry by interfering with angiotensin-converting enzyme 2 (ACE2) receptors, (2) viral replication, (3) the rapid exacerbation of inflammation to a likely fatal hyperinflammatory cytokine storm related to the toll-like receptor 4 (TLR4) pathway and NLR family pyrin domain containing 3 (NLRP3) inflammasome, and (4) oxidative stress. On the one hand, H_2_S protects against SARS-CoV-2; on the other hand, it is known that the virus itself downregulates the production of H_2_S by reducing the expression of CBS, CSE/CTH, and 3-MST enzymes, a fact that favors viral replication [10]. Regarding the first point, it is known that SARS-CoV-2 infection determines the downregulation of the ACE2 receptor [11,12]. However, ACE2 is recognized as a vasodilating, anti-inflammatory, antioxidant, and antifibrotic factor by cleaving Angiotensin (Ang) II into Ang (1–7) and, hence, NO production via NO synthase [13]. Accordingly, the relatively increased Ang II reduces NO synthesis [12] and, in addition, triggers nicotinamide adenine dinucleotide phosphate (NADPH) oxidase with the generation of superoxide (O^2−^) radicals, which have a proinflammatory impact [14,15]. H_2_S has been reported to induce ACE2 upregulation, reducing organ and especially pulmonary tissue injuries related to oxidative stress and inflammation [16]. Furthermore, H_2_S interferes with transmembrane protease serine 2 (TMPRSS2), a protease that boosts the entry of SARS-CoV-2 via the ACE2 receptor [17].

Like the effect of H_2_S on the replication of a series of RNA viruses [18,19,20,21,22], H_2_S was recently reported to regulate SARS-CoV-2 infection by keeping virus replication under control [10].

As for the activity of H_2_S on TLR4/NF-kB activity related to SARS-CoV-2 infection, it is known that SARS-CoV-2 can intensify IkB removal from NF-kB, resulting in NF-kB activation and hence extraordinary cytokine secretion [23,24]. In this context, H_2_S has been found to inhibit NF-kB activation through persulfidation of IkB tied up to NF-kB [25]. Furthermore, H_2_S has been reported to suppress the activity of the NLRP3 inflammasome and hence further diminish proinflammatory cytokine generation [26,27].

As for oxidative stress, it has been reported that SARS-CoV-2 infection lowers the expression of CBS, CSE/CTH, and 3-MST and thus H_2_S synthesis [10]. Since H_2_S has been reported to contribute strongly to mitochondrial bioenergetics [28,29], its reduction limits mitochondrial oxidative phosphorylation (OXPHOS), which implies TCA cycle activity and mitochondrial respiration inhibition with secondary production of reactive oxygen species (ROS) [28,29]. Furthermore, RNA-seq demonstrated that depletion of H_2_S caused by SARS-CoV-2 lowers GSH synthesis [10] and reduces Nrf2 gene expression, hence their antioxidant potential [10]. In turn, oxidative stress has been shown to inhibit mitochondrial 3-MST activity and interfere with the positive bioenergetic role of the enzyme, which produces further reduction in H_2_S and worsening of oxidative stress [30]. However, it is known that different forms of cellular stress can induce the upregulation of CSE [31,32,33,34,35,36] and that, among the different stressors, oxidative stress might play a major role in triggering CSE as a sort of homeostatic response promoting the generation of different antioxidant molecules, including GSH and H_2_S [2].

N-acetylcysteine (NAC), a precursor of GSH which possesses potent antioxidant and anti-inflammatory attributes [9], has been shown to ameliorate immunity, decrease inflammation, and suppress viral replication in COVID-19 [37,38,39]. In addition, Ezerina et al. [40] reported that the antioxidant properties of NAC could also be ascribed to the synthesis of H_2_S and other sulfane/sulfur compounds. Accordingly, considering the known effects of H_2_S on COVID-19 infection [9,10] and the fact that the serum concentration of H_2_S was found to be low in patients with severe infection [41], NAC could be beneficial in treating these patients. In this context, a recent meta-analysis based on 11 out of 12 randomized clinical trials reported that NAC decreased mortality in COVID-19 patients by about 41%. At the same time, 6 out of 12 demonstrated an improvement in the recovery/discharge ratio [42].

Therefore, in this study, we first assessed specific clinical and biochemical characteristics in hospitalized non-survivor and survivor patients affected by COVID-19 pneumonia with acute respiratory distress syndrome compared to non-COVID-19 subjects. Then, we focused on measuring plasma H_2_S and malondialdehyde (MDA), a reliable marker of oxidative stress, as indicators of in-hospital mortality observed within 30 days of admission. Finally, we analyzed whether oxidative stress is a true stressor for H_2_S synthesis and whether NAC can increase H_2_S and GSH concentrations in cultured human small airway epithelial cells (SAECs) and monocyte-like THP-1 cells.

## 2. Materials and Methods

### 2.1. Study Population and Design

In total, 80 COVID-19 patients (40 survivors and 40 non-survivors) with clinical features and imaging consistent with COVID-19 pneumonia who were hospitalized at Verona University Hospital (Italy) from April 2020 to May 2021 [43] and 40 age/sex-matched non-COVID-19 subjects were enrolled in this study. This study was approved by the Ethics Committee of the Azienda Ospedaliera Universitaria Integrata Verona (prot. n. 3982CESC). Oral informed consent with annotation in the medical records was considered sufficient for the Ethical Committee. Written informed consent was acquired from all the non-COVID-19 subjects before their enrolment. In COVID-19 subjects, demographic characteristics, past medical history, biochemical parameters, and arterial blood gas test results were collected at hospital admission. Information about in-hospital evolution was retrospectively collected from the medical records. No patients enrolled in the study received NAC during hospitalization.

### 2.2. Plasma Sample Collection and Biochemical Parameter Measurement

In this study, we used the plasma samples of hospitalized COVID-19 patients stored at −80° C in an authorized biobank. Blood samples were obtained within 24 h of hospital admission (to avoid confounding factors related to the subsequent treatments). Plasma samples of all the patients enrolled in this study were collected after whole blood centrifugation at 400× *g* for 10 min. The undiluted plasma was then transferred to 10 mL polypropylene tubes, aliquoted, and stored at −80 °C for subsequent analysis. Laboratory assessments comprised complete blood count, iron metabolism biomarkers, liver and renal function, and C-reactive protein.

### 2.3. MDA and H_2_S Plasma Measurement

As for the evaluation of oxidative stress markers, in this study, we assessed the concentration of plasma MDA, which was measured using commercial high-performance liquid chromatography (HPLC) kits provided by Chromsystems (Munich, Germany). The preparation of the samples was carried out according to the manufacturer’s instructions and was based on an effective protein precipitation step followed by derivatization. The chromatographic separation was run on an isocratic HPLC system Waters Alliance e2695 (Alliance, Waters Corporation, Milford, MA, USA) with a fluorescence detector (2475 FLR Detector) (Alliance, Waters Corporation, Milford, MA, USA).

H_2_S in plasma of all the patients included in the study was measured using a very sensitive method of measuring the presence of H_2_S down to nanomolar levels, using monobromobimane (MBB). This method involves the derivatization of sulfide with excess MBB in 100 mM Tris–HCl buffer (pH 9.5, 0.1 mM DTPA) for 30 min in 1% oxygen at room temperature. The fluorescent product sulfide-dibimane (SDB) is analyzed by reverse-phase (RP)-HPLC by using Waters Alliance e2695 (Alliance, Waters Corporation, Milford, MA, USA) with a fluorescence detector (2475 FLR Detector) (Alliance, Waters Corporation, Milford, MA, USA), as previously described [44]. We chose this method because it is suitable for the sensitive quantitative measurement of free H_2_S in multiple biological samples such as plasma, tissue and cell culture lysates, or media.

### 2.4. Cell Cultures

Human monocytic leukemia cells THP-1 (AddexBio, San Diego, CA, USA) were expanded and selected based on previously obtained evidence [45,46,47]. SAECs were also utilized and cultured following the recommended manufacturer’s protocols (ScienceCell, Carlsbad, CA, USA). Endotoxin contamination of cell culture has been routinely excluded with the chromogenic Limulus amebocyte lysate assay.

### 2.5. Cell Viability Assay

It is widely acknowledged that the quantification of cell viability serves as the basis for a plethora of in vitro assays that assess cellular responses to external stimuli [48,49]. Cellular viability was evaluated with a PE Annexin V Apoptosis Detection Kit (BD Biosciences, Franklin Lakes, NJ, USA) as previously reported [50,51,52,53]. The fluorescence intensity of cells per sample was measured by flow cytometry using the BD FACS Canto cytofluorometer. A minimum of 10,000 cells were analyzed via flow cytometry, and quantitative analysis was performed in FlowJo (BD Biosciences, Franklin Lakes, NJ, USA). All the assays were performed in triplicate.

### 2.6. Intracellular ROS Measurement

A CellROX Deep Red Flow Cytometry Assay Kit (Molecular et al., Carlsbad, CA, USA) was used for the determination of intracellular ROS formation [46,54]. As previously described in [53], cells were seeded in 24-well plates at a density of 5 × 10^5^ cells/mL. To explore the effect of NAC on counteracting oxidative stress, increasing concentrations (from 0.04 to 5 mM) of NAC were added to the cells overnight. Then, to induce oxidative stress, THP-1 cells and SAECs were incubated with 200 μM tert-butyl hydroperoxide (TBHP) for 45 min at 37 °C. After incubation of the cells with TBHP, the CellROX Deep Red reagent at a final concentration of 500 nM was added to the cells for 45 min at 37 °C and then immediately analyzed via flow cytometry using the FACS BD LSR Fortessa X-20 cytofluorometer (BD Biosciences, Franklin Lakes, NJ, USA).

### 2.7. Determination of Cellular Lipid Peroxidation

Lipid peroxidation was measured using C11-BODIPY (581/591) (Life Technologies, Grand Island, NY, USA), as detailed in reference [55,56]. Cells were seeded in 24-well plates at a density of 5 × 10^5^ cells/mL. After 24 h, to determine the impact of NAC on lipid peroxidation, increasing concentrations of NAC (ranging from 0.04 to 1 mM) were added to THP-1 cells and SAECs overnight, before the addition of TBHP. Following this, the cells were stained with 2 μM BODIPY for 30 min at 37 °C and promptly analyzed via flow cytometry.

### 2.8. Measurement of GSH and GSSG

GSH and GSSG were measured in both plasma and cell line samples following the method of Enomoto et al. [57], by using an Agilent Technologies 1260 Infinity System (Agilent Technologies, Santa Clara, CA, USA) consisting of an autosampler (G1367E), pump (G1311B), and column compartment (G1316A) connected to an Agilent Technologies 6460 triple quadrupole mass spectrometer (Agilent Technologies, Santa Clara, CA, USA)**.**

### 2.9. Statistical Analysis

Data are expressed as mean ± standard deviation (SD) values or median ± interquartile range if normally distributed. The normality of the data distribution was assessed using the Shapiro–Wilk test [58]. The test revealed that only a few parameters followed a normal distribution. Given the non-normal distribution of most of the data, the Wilcoxon test [58] was employed to compare differences between survivor and non-survivor patient groups across demographic, clinical, and biochemical characteristics. In addition, the effect sizes were assessed using Cohen’s d method for MDA and H_2_S to determine the magnitude of relationship between groups, and a correlation matrix between several parameters was performed. Kaplan–Meier (KM) survival curves were constructed for each clinical and biochemical feature to explore their univariate association with mortality. Patients were dichotomized in high vs. low groups based on the median value of the parameter under consideration, and for H_2_S, tertiles were also tested. The log-rank test (Mantel–Cox) was used to assess differences in survival curves between the two groups. Multivariate Cox regression was performed to determine if H_2_S is an independent predictor. Model performance metrics, AUC, sensitivity, and specificity were calculated for H_2_S as mortality predictors. A significance threshold of 0.05 was applied for determining statistical significance. A statistical analysis was performed using SPSS^®^ version 27 (IBM) and R version 4.3 (R Foundation for Statistical computing, Vienna, Austria).

## 3. Results

### 3.1. Clinical and Biochemical Parameters in the Three Groups of Subjects Participating in the Study

According to the inclusion specifications, 80 hospitalized patients (40 survivors and 40 non-survivors), affected by COVID-19 pneumonia and subsequent acute respiratory insufficiency, and 40 non-COVID-19 subjects were recruited in this study. The mean interval from the beginning of COVID-19 symptoms to hospital entry was shorter in non-survivors than in survivors (*p* < 0.05). Respiratory insufficiency expressed as the ratio of arterial oxygen partial pressure (PaO_2_) to the fraction of inspired oxygen (FiO_2_) (P/F) was more severe in non-survivor patients than survivor patients (*p* < 0.0001). All the patients needed an additional supplement of O_2_, which was given in different ways during hospitalization. Entry to the Intensive Care Unit and the requirement for orotracheal intubation were greater in non-survivors than in survivor patients (*p* < 0.001). Following the inclusion specifications, the demographic features were comparable in the three groups. In addition, most of the patients had at least one comorbidity, with hypertension and diabetes mellitus being more highly represented in all the three groups. This evidence indicates that comorbidities had no noteworthy influence on the clinical sequelae of our patients (Table S1). Biochemical parameters such as creatinine and alanine aminotransferase (ALT) were comparable and within normal limits in the three groups. Aspartate aminotransferase (AST) and CRP concentrations were elevated in both COVID-19 groups (*p* < 0.001) (Table S1).

Amid iron metabolism variables, ferritin was comparably augmented, while iron, transferrin, and transferrin saturation were almost identically reduced in COVID-19 groups, when juxtaposed with non-COVID-19 subjects (Table S2). As for blood parameters, hematocrit, hemoglobin, platelets, neutrophils, monocytes, basophils, and lymphocytes were lower in all COVID-19 patients than in non-COVID-19 subjects. Red cell distribution scattering was higher in the non-survivor group than in the survivor group (Table S2). A matrix correlation heatmap between several clinical and biochemical parameters of COVID-19 patients has been added in the Supplementary File (Figure S1). We did not find a significant correlation between H_2_S, MDA, P/F ratio, CRP, ferritin, and mortality. However, we must emphasize that our data were obtained at hospital admission, and as is known, the clinical evolution in COVID-19 patients has often been independent of the initial clinical presentation.

#### Plasma Concentrations of MDA and H_2_S

**Figure 1:**
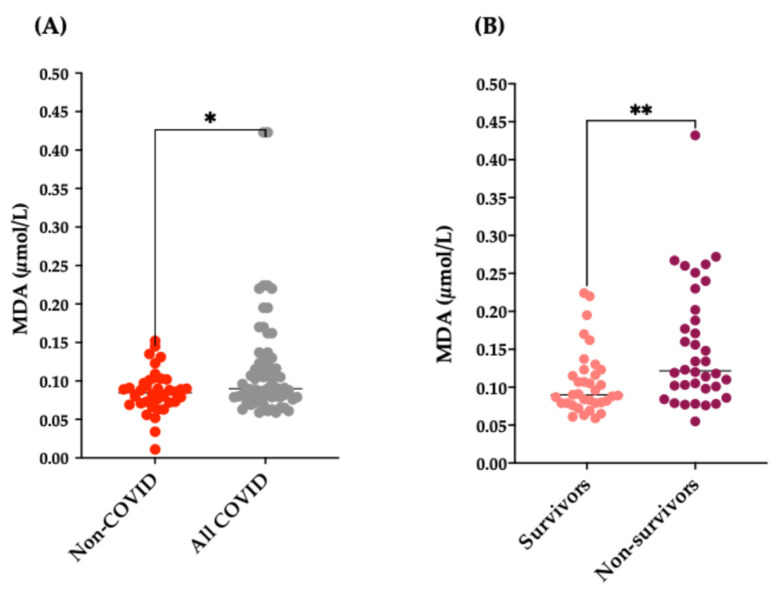
. MDA plasma levels in all three groups of subjects. (**A**) MDA levels in non-COVID-19 subjects and in all COVID-19 patients; * *p* = 0.03 vs. non-COVID. (**B**) MDA levels in survivor and non-survivor COVID-19 patients; ** *p* = 0.0043 vs. non-survivors.

As for the evaluation of oxidative stress markers, in this study, we evaluated the concentration of plasma MDA. MDA levels were lower in non-COVID-19 subjects than in the two groups of COVID-19 patients taken together (Figure 1A; *p* = 0.03). Intriguingly, MDA plasma concentrations were significantly higher in non-survivor COVID-19 patients than in survivors (Figure 1B; *p* = 0.0043). MDA demonstrated a medium effect size (Cohen’s d = −0.688, 95% CI: −1.146/−0.230) in distinguishing between deceased and not deceased, with higher concentrations of MDA related to negative outcome (Table S3).

**Figure 2:**
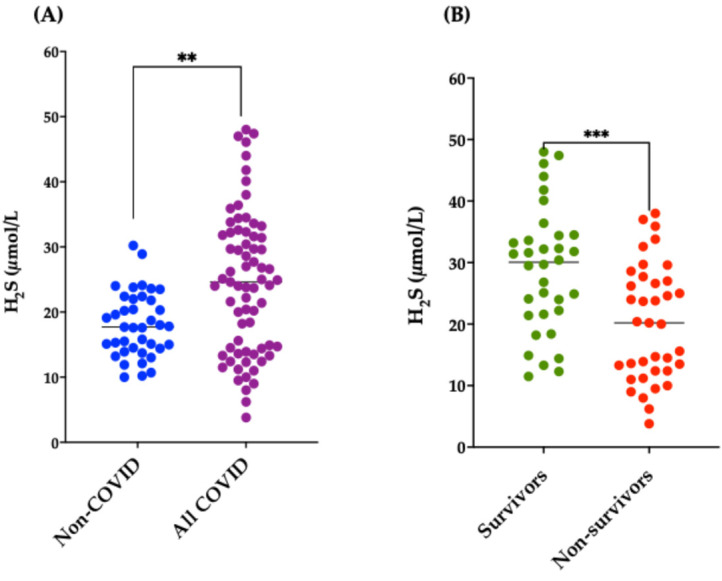
H_2_S Plasma levels in all the participants in the study. H_2_S plasma levels in all three groups of subjects. (**A**) H_2_S plasma levels in non-COVID-19 subjects and in all COVID-19 patients; ** *p* = 0.0016 vs. non-COVID. (**B**) H_2_S plasma levels in survivor and non-survivor COVID-19 patients; *** *p* = 0.0008 vs. non-survivors.

Subsequently, we evaluated H_2_S in the plasma of all the subjects participating in the study. As shown in Figure 2A, H_2_S levels were significantly increased in all COVID-19 patients (both survivors and non-survivors) compared to non-COVID-19 (*p* = 0.0016); however, when we evaluated H_2_S levels separately in COVID-19 patients (Figure 2B), we found that the survivor COVID-19 patients had significantly higher plasma levels than non-survivors (*p* = 0.008). H_2_S demonstrated a medium effect size (Cohen’s d = 0.744, 95% CI: 0.284/1.204) in distinguishing between deceased and not deceased, with higher concentrations of H_2_S related to positive outcomes (Table S3).

**Figure 3:**
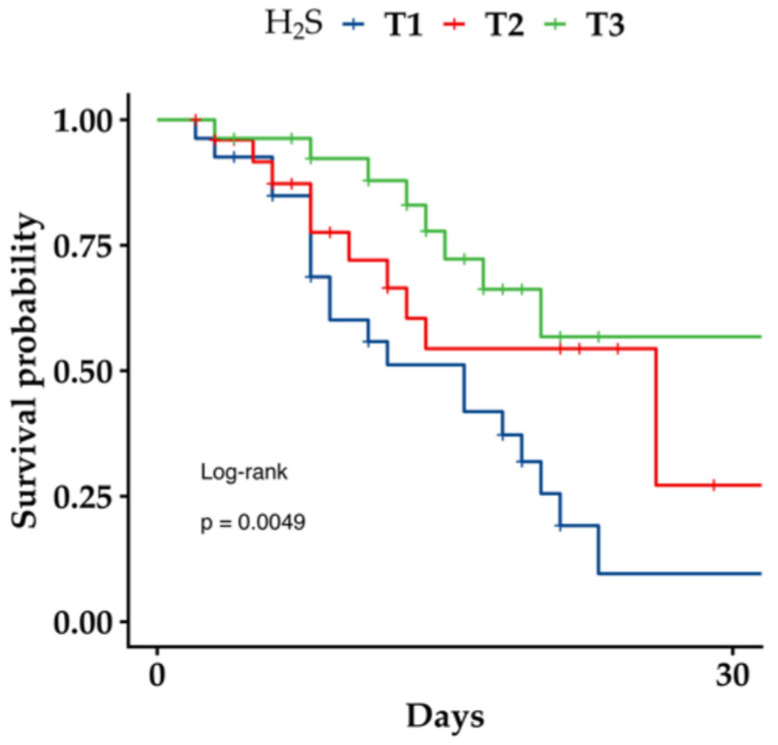
Survival curves in COVID-19 patients in relation to tertiles of plasma concentrations of H_2_S. Kaplan–Meier curves showing survival at 30 days in COVID-19 patients.

Then, we evaluated the univariate association of H_2_S and MDA with patient survival over time using Kaplan–Meier curves. For each variable, patients were stratified into high vs. low groups based on the median baseline value. There was a trend showing a reduced survival probabilities in patients with higher MDA levels, but the log-rank did not reach statistical significance (log-rank *p* = 0.076, Figure S2A). In contrast, the Kaplan–Meier curve for H_2_S highlighted markedly reduced survival probabilities in patients with lower H_2_S levels (log-rank *p* = 0.004) (Figure S2B). In addition, when dividing the H_2_S variable into tertiles (Figure 3), a significant dependence on concentration was observed (log-rank *p* = 0.0049).

The ROC analysis for H_2_S demonstrates a fair ability to predict mortality (AUC = 0.693), which is moderately better than chance predictions but below the threshold for good performance. The test shows strong specificity (82.5%), indicating reliable identification of true negative cases, and reasonable positive predictive value (74.1%). Overall, the test has moderate diagnostic accuracy and is more suitable as an “exclusion” tool given its high specificity (Figure S3). Subsequently, we perform a multivariate Cox regression to understand which covariates potentially affect patient prognosis (Table 1). Our results confirm that age and severity of respiratory failure are significant predictors of survival. More interestingly, they also show that plasma H_2_S is a significant predictor of survival, whereas comorbidities and other biochemical parameters are not.

### 3.2. In Vitro Study

#### 3.2.1. Effect of Oxidative Stress on H_2_S Synthesis in THP-1 Cells

**Figure 4:**
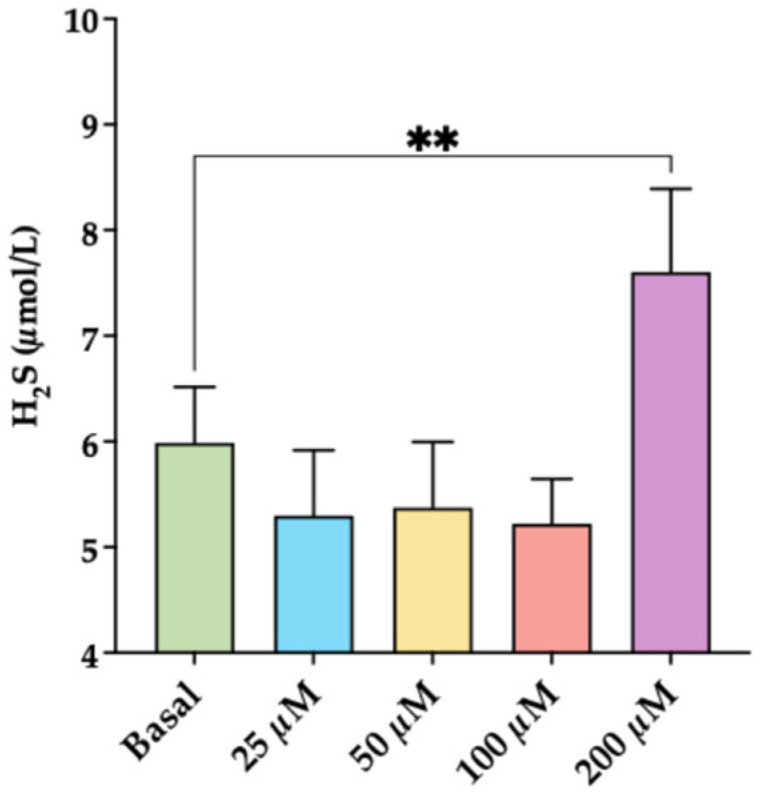
Effect of increasing concentration of TBHP on H_2_S formation. Cells were incubated with increasing concentrations (from 25 to 200 μM) of tert-butyl hydroperoxide for 45 min. Data represent the mean ± SD of measurements performed in triplicate in three different experiments. ** *p* < 0.01.

To first evaluate whether oxidative stress is a true stressor for H_2_S synthesis, we assessed the effect of increasing concentrations of TBHP on H_2_S concentration in THP-1 cells. Figure 4 shows that, at the highest concentrations, TBHP significantly increased the generation of H_2_S (*p* < 0.01).

#### 3.2.2. Cell Viability of THP-1 Cells and SAECs Incubated with NAC

**Figure 5:**
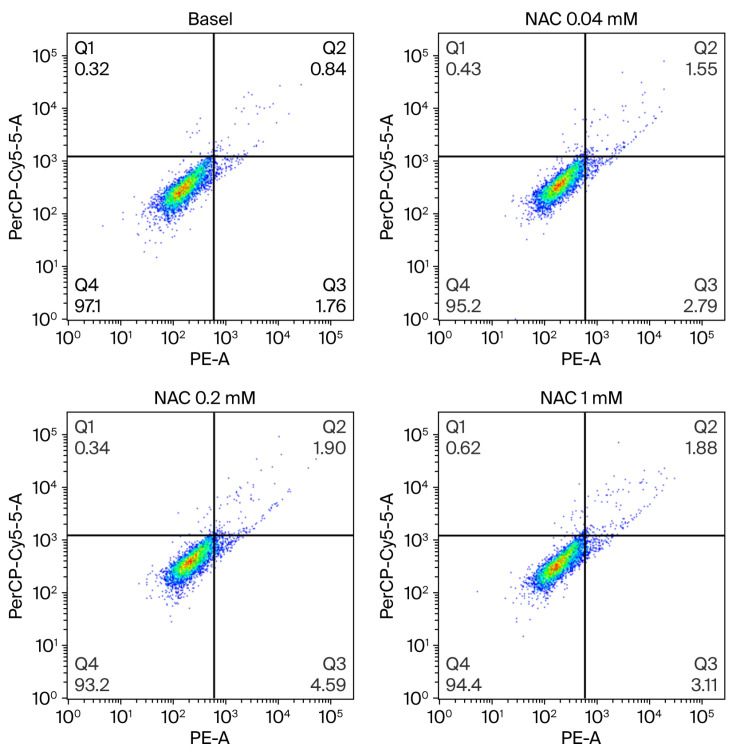
Effect of increasing concentration of NAC on SAEC cell viability. NAC did not affect cell viability in small airway epithelial cells (SAECs). Representative flow cytometry analysis of different concentrations of NAC on SAEC viability. Cells were incubated overnight with increasing concentrations of NAC (from 0.04 to 1 mM). Q4: live cells; Q3: pre-apoptotic cells; Q2: apoptotic cells; Q1: necrotic cells.

Then, we performed experiments to assess the effect of increasing NAC concentration (from 0.04 mM to 1 mM) on THP-1 and SAEC cell viability when incubated overnight. Since our results indicate that NAC did not affect the number of necrotic, pre-apoptotic, and apoptotic cells in both cell lines, the subsequent experiments were performed using these concentrations. Figure 5 shows a representative flow cytometry analysis of dose–response NAC on SAEC cell viability.

#### 3.2.3. Effect of NAC on Intracellular Oxidative Stress

**Figure 6:**
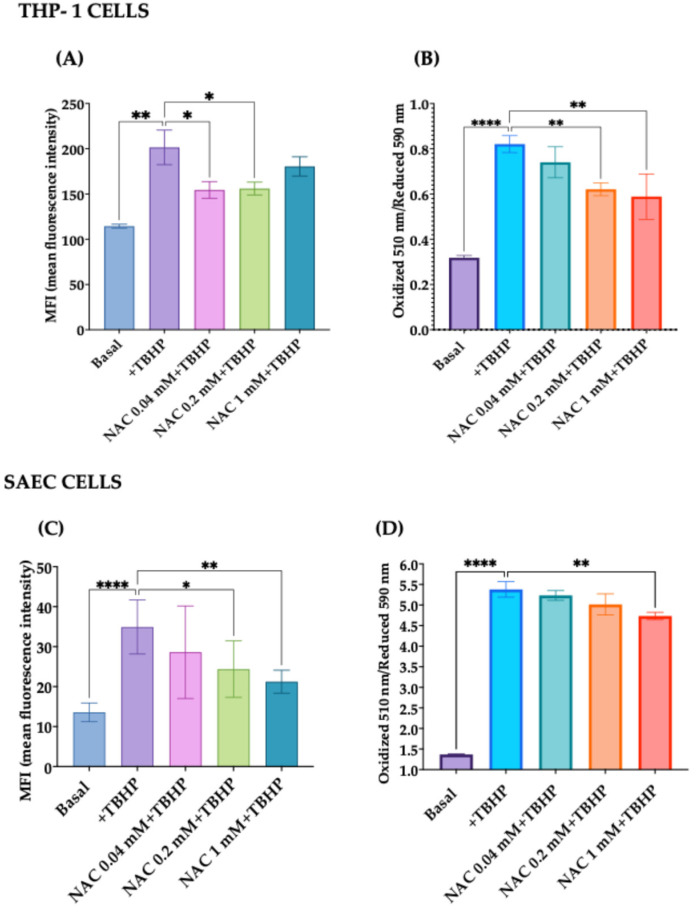
Inhibitory effects of NAC on intracellular ROS formation and lipid peroxidation in THP-1 cells and SAECS. (**A**) Dose-dependent inhibitory effect of NAC on intracellular ROS levels in THP-1, expressed as fluorescence intensity (MFI). (**B**) Dose-dependent effect of NAC in mitigating lipid peroxidation in THP-1 cells. Results are expressed as the ratio of oxidized dye fluorescence (510 nm) and reduced dye fluorescence (590 nm). (**C**) NAC-induced reduction in ROS formation in SAECs. (**D**) Dose-dependent effect of NAC in mitigating lipid peroxidation in SAECs. Data represent the mean ± SD of measurements performed in triplicate in three different experiments. * *p* < 0.05; ** *p* < 0.01; **** *p* < 0.0001.

To evaluate the possible antioxidant effect of NAC on intracellular ROS formation, we preincubated THP-1 cells and SAECs with increasing concentrations of NAC (from 0.04 mM to 1 mM) overnight, before the addition of TBHP. Our results show that NAC dose-dependently reduced intracellular ROS formation starting from NAC 0.04 mM in both cell lines (*p* < from 0.05 to 0.01), as shown in Figure 6A,C.

Subsequently, we assessed whether NAC could also affect lipid peroxidation. Our results show a dose-dependent significant reduction in lipid peroxidation starting from NAC 0.2 mM in SAECs (*p* < 0.01), whereas this effect in THP-1 was evident only with the highest concentration (Figure 6B,D).

#### 3.2.4. Dose–Response Effect of NAC on Cellular GSH, GSH/GSSG Ratio, and H_2_S

**Figure 7:**
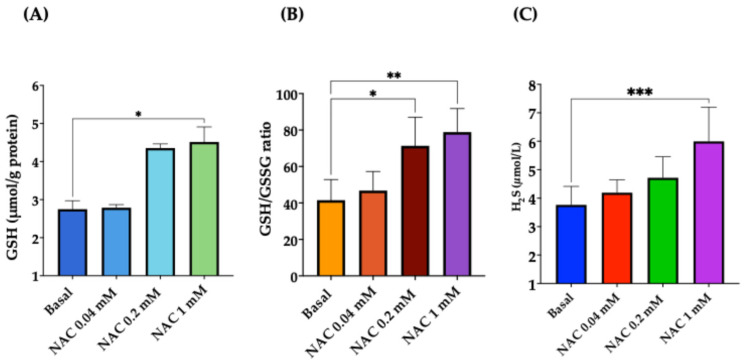
Dose–response effect of NAC on cellular GSH, GSH/glutathione oxidized (GSSG) ratio, and H_2_S. THP-1 cells were preincubated overnight with increasing concentrations (from 0.04 to 1 mM) of NAC. (**A**) Dose–response effect of NAC on GSH concentration. (**B**) Dose–response effect of NAC on GSH/GSSG ratio. (**C**) Dose–response effect of H_2_S concentrations. Data represent the mean ± SD of measurements performed in triplicate in three different experiments. * *p* < 0.05; ** *p* < 0.01; *** *p* < 0.001.

Furthermore, we investigated whether NAC affects GSH and GSSG concentrations in both cell lines. Our results show that NAC 1 mM significantly increased the GSH concentration (*p* < 0.01). However, and most interestingly, NAC led to a dose-dependent significant rise in the GSH/GSSG ratio in both SAECs and THP-1 cells (Figure 7A,B).

Finally, we evaluated the effect of NAC (from 0.4 to 1 mM) on the cellular H_2_S concentration. Figure 7C shows that NAC led to a dose-dependent increase in H2S, which became significant at the highest concentration (*p* < 0.01).

## 4. Discussion

H_2_S has been reported to act against COVID-19 infection, particularly by inhibiting viral cell entry, replication, oxidative stress, and the escalation of inflammation that promotes a cytokine storm [10]. Following these observations indicating that H_2_S may play a key role in the pathogenesis of COVID-19, we evaluated H_2_S in the plasma of survivor and non-survivor patients with COVID-19 pneumonia compared with age- and sex-matched non-COVID-19 subjects. Unexpectedly, COVID-19 patients had higher H_2_S than non-survivors and controls. A previous clinical study proposed an inverse relationship between endogenous H_2_S levels and the severity of COVID-19 [59]. In contrast, and in agreement with our results, Renieris et al. found that survivors, unlike non-survivors, had significantly higher H_2_S levels on days 1 and 7 after admission [42]. Based on the present data, we cannot fully explain why survivors have higher levels of H_2_S than non-survivors and non-COVID-19 subjects, a fact that contradicts initial mechanistic assumption.

However, we present a possible explanation as to why COVID-19 patients who survived have higher H_2_S plasma levels than non-survivors and non-COVID-19 subjects. It is known that CSE is constitutively expressed in many cell types and that different forms of cellular stress can induce the upregulation of the enzyme [32,33,34,35,36,37]. Among the various stressors, oxidative stress may play a significant role in triggering CSE and other enzyme expression as a form of homeostatic response, promoting the generation of different antioxidant molecules, including GSH and H_2_S, and thereby balancing oxidative stress [2]. Interestingly, our in vitro study confirms that oxidative stress is a valid stressor for H_2_S synthesis. However, it is known that SARS-CoV-2 can inhibit the expression of CSE, CBS, and 3-MST, and hence H_2_S synthesis [11], which may in some way mitigate the stressor effect of oxidative stress. Accordingly, lower levels of H_2_S may disrupt the cellular redox state by reducing TCA cycle activity and mitochondrial respiration, leading to the secondary production of ROS [29,30]. In addition, the downregulation of the ACE2 receptor caused by SARS-CoV-2 is only partially offset by the lower activity of H_2_S, which increases Ang II and, consequently, NADPH oxidase, leading to the generation of O^2−^ radicals [15,16]. Finally, depletion of H_2_S lowers GSH synthesis [11] and reduces Nrf2 gene expression, thereby decreasing their antioxidant potential [11]. Therefore, the inhibition of CSE, CBS, and 3-MST induced by SARS-CoV-2 may exacerbate the onset and progression of oxidative stress, and the H_2_S-inducing stressors may only partially counterbalance this effect. In fact, this study found that oxidative stress, measured as circulating MDA, was higher in non-survivors than in survivors, who had oxidative stress levels similar to those of non-COVID-19 subjects. These results may help explain why H_2_S was higher in survivors than in non-survivors who had H_2_S levels like those of non-COVID-19 subjects. Therefore, we speculate that the increased oxidative stress found in non-survivors may be related to an almost complete inhibition of CSE, CBS, and 3-MST induced by SARS-CoV-2 and not adequately counteracted by the action of cellular stressors. In contrast, in survivors, there may be an inferior inhibition of the H_2_S-generating enzymes, parallel to an appropriate activity of stressors, such as oxidative stress and inflammation. Even if, based on the present results, we cannot establish what determines the degree of CSE, CBS, and 3-MST inhibition, it may likely depend on viral load. Furthermore, oxidative stress is known to inhibit 3-MST [31], a fact that further contributes to reducing H2S synthesis in non-survivors. It is essential to note, however, that our study did not evaluate the activity of H_2_S-generating enzymes; therefore, further studies are needed to support this conclusion fully. Moreover, the higher oxidative stress in non-survivors can also reduce H_2_S, as it has been shown that H_2_S can react with various ROS, such as superoxide, at somewhat higher rates than other classic antioxidants, such as cysteine and GSH [2]. Overall, the results of this study open up the possibility of future significant research to understand why certain people respond by producing more H_2_S while others do not.

There may be alternative explanations that account for or contribute to the increase in H_2_S in survivors of COVID-19. In particular, inflammatory stimuli and hypoxia, mediated by hypoxia-inducible factor-1, are known to upregulate CSE [59,60,61]. In addition, another possibility is that survivors may mount a stronger response through so far unknown mechanisms. Interestingly, the results of this study also show that the Kaplan–Meier curve for H_2_S indicates a markedly reduced survival probability in patients with lower H_2_S levels. These findings confirm the important prognostic role of H_2_S at hospital admission and demonstrate the most robust association with survival in the log-rank test, stratifying patients into different groups based on their tertile levels. These curves illustrate how survival probabilities diverge over time, reinforcing the prognostic value of H_2_S. Similarly, there was a trend of MDA showing reduced survival probabilities in patients with higher oxidative stress. Accordingly, the reduced survival probabilities of patients with a lack of H_2_S response to SARS-CoV-2, such as non-survivors, may be related to the well-documented effects of H_2_S on viral entry, replication, TMPRSS2 activity, inflammation, and oxidative stress [10].

The results of this study also confirm that NAC possesses potent antioxidant properties since it reduces ROS formation and lipid peroxidation in cultured cells stimulated with TBHP. Furthermore, NAC increased GSH and ameliorated the GSH/GSSG ratio. Most importantly, in this study, and in agreement with Ezerina et al. [41], NAC was shown to increase H_2_S synthesis in TPH-1 cells. Since 11 out of 12 randomized clinical trials have shown that NAC reduces mortality in COVID-19 patients by approximately 41%, and at the same time, 6 out of 12 demonstrated an improvement in the recovery/discharge ratio [43], this molecule might likely be helpful in all the clinical situations characterized by low levels of H_2_S or inability to produce an adequate H_2_S response like in SARS-CoV-2 patients. It is essential to emphasize that we evaluated the effect of NAC on H_2_S in cultured cells, and the possibility that this translates to clinical benefit requires prospective trials. Therefore, based on the current results, we do not recommend NAC broadly without the support of randomized clinical trials.

### 4.1. Limitations of the Study

In this study, there are several limitations that dampen the conclusions. First of all, the study was conducted at a single center with only one time point measured. Furthermore, this study did not include a validation cohort. In particular, the inability to follow H_2_S plasma concentrations over the course of the disease limits the conclusions of our study. Secondly, in this study, we did not measure the activity of H_2_S-generating enzymes, and therefore, at least a part of conclusions should be taken with caution, and further studies on this topic are needed to fully unravel our hypothesis. Furthermore, in this study, we were unable to account for all treatments received by the COVID-19 patients before entering the hospital. It is important to note that our findings apply to the original strain of COVID-19. Currently, morbidity and mortality associated with viral strains are significantly lower; therefore, the described associations may no longer be valid. As for the cell culture model, it is unlikely that the positive effects of NAC on H_2_S in this model can simply translate into clinical benefits since the model does not replicate viral infection in vivo, and further clinical studies are needed to extend our results to clinical practice.

### 4.2. Clinical Implications

Results from the preclinical studies show both antiviral and anti-inflammatory features of H_2_S (reviewed in [10]). In particular, infected respiratory epithelial cells revealed a decreased capacity to produce endogenous H_2_S but an increased tendency to deteriorate H_2_S, suggesting that viral infection may affect H_2_S homeostasis (reviewed in [10]). Thus, H_2_S measurement provides a rationale for testing risk stratification for viral infections and other diseases, as well as for initiating therapeutic H_2_S modulation. Of course, therapeutic H_2_S modulation requires prospective studies in different diseases using NAC or other molecules generating H_2_S, measuring H_2_S as the mechanistic endpoint.

## 5. Conclusions

In conclusion, the results of this study suggest that, in non-survivor patients affected by COVID-19, there may be a lack of homeostatic response that promotes the generation of antioxidant molecules, including GSH and H_2_S. In other words, we hypothesize that, in non-survivors, the usual stressors, like oxidative stress and inflammation habitually inducing the generation of H_2_S and GSH, may be counterbalanced by the potent inhibition of H_2_S-generating enzymes caused by SARS-CoV-2. The epiphenomenon of this inhibition is the increase in oxidative stress, as evidenced by the increase in circulating MDA in non-surviving patients. The reduced availability of H_2_S is likely to decrease TCA cycle activity and increase NADPH oxidase activity, resulting in augmented production of ROS. In turn, the increased oxidative stress can further reduce H_2_S by inhibiting 3-MST and because H_2_S can react with various ROS, such as superoxide. In survivors, the balance between the inhibition of H_2_S by SARS-CoV-2 and the induction of H_2_S by stressors such as oxidative stress and inflammation remains in favor of H_2_S production. The fact that there is no increased oxidative stress in these patients confirms this hypothesis. Interestingly, the Kaplan–Meier curve for H_2_S indicates a markedly reduced survival probability in patients with lower H_2_S levels, reinforcing the prognostic value of H_2_S. Finally, the results of this study suggest that NAC may be beneficial in all clinical situations characterized by low levels of H_2_S or an inadequate H_2_S response, such as in COVID-19 patients. Future prospective studies using NAC or other molecules generating H_2_S should measure H_2_S as a mechanistic endpoint.

## Figures and Tables

**Table 1:** Multivariate cox regression in COVID-19 patients.

Characteristic	HR	95% CI	*p*-Value
**H_2_S**	0.95	0.91, 0.99	**0.010**
**P/F ratio**	0.99	0.99, 1.00	**0.009**
**Age**	1.10	1.03, 1.17	**0.004**
**Comorbidities**			
False	-	-	
True	0.54	0.25, 1.16	0.11
**Platelets**	1.00	1.00, 1.00	0.15
**Creatinine**	0.93	0.87, 0.99	0.052
**AST**	1.01	0.99, 1.04	0.2
**Lymphocytes**	0.72	0.23, 2.27	0.6

Abbreviations: CI = Confidence Interval; HR = Hazard Ratio.

## Data Availability

The original contributions presented in this study are included in the article/Supplementary Material. Further inquiries can be directed to the corresponding author.

## Supplementary Materials

The following supporting information can be downloaded at: https://www.mdpi.com/article/10.3390/antiox15030307/s1. Table S1: Anthropometric, clinical characteristics and routine laboratory parameters in the three groups of subjects participating in the study. Table S2: Iron metabolism parameters and complete blood count in the three groups of subjects participating in the study. Table S3: Effect sizes with Cohen’s d Method for H_2_S and MDA. Figure S1: Correlation heatmap between several clinical and biochemical parameters. Figure S2: Survival curves for median plasma concentration of MDA (A) and H_2_S (B). Figure S3: ROC curve analysis for H_2_S as mortality predictor.

## References

[1] Weekly Epidemiological Update on COVID-19-1 September 2023. https://www.who.int/publications/m/item/weekly-epidemiological-update-on-covid-19---1-september-2023.

[2] Cirino G., Szabo C., Papapetropoulos A. (2023). Physiological Roles of Hydrogen Sulfide in Mammalian Cells, Tissues, and Organs. Physiol. Rev..

[3] Chiku T., Padovani D., Zhu W., Singh S., Vitvitsky V., Banerjee R. (2009). H2S Biogenesis by Human Cystathionine Gamma-Lyase Leads to the Novel Sulfur Metabolites Lanthionine and Homolanthionine and Is Responsive to the Grade of Hyperhomocysteinemia. J. Biol. Chem..

[4] Singh S., Padovani D., Leslie R.A., Chiku T., Banerjee R. (2009). Relative Contributions of Cystathionine β-Synthase and γ-Cystathionase to H2S Biogenesis via Alternative Trans-Sulfuration Reactions. J. Biol. Chem..

[5] Sun X., Wu S., Mao C., Qu Y., Xu Z., Xie Y., Jiang D., Song Y. (2024). Therapeutic Potential of Hydrogen Sulfide in Ischemia and Reperfusion Injury. Biomolecules.

[6] Zhu C., Liu Q., Li X., Wei R., Ge T., Zheng X., Li B., Liu K., Cui R. (2022). Hydrogen Sulfide: A New Therapeutic Target in Vascular Diseases. Front. Endocrinol..

[7] Abdulle A.E., Van Goor H., Mulder D.J. (2018). Hydrogen Sulfide: A Therapeutic Option in Systemic Sclerosis. Int. J. Mol. Sci..

[8] Yang J., Minkler P., Grove D., Wang R., Willard B., Dweik R., Hine C. (2019). Non-Enzymatic Hydrogen Sulfide Production from Cysteine in Blood Is Catalyzed by Iron and Vitamin B6. Commun. Biol..

[9] Bourgonje A.R., Offringa A.K., Van Eijk L.E., Abdulle A.E., Hillebrands J.-L., Van Der Voort P.H.J., Van Goor H., Van Hezik E.J. (2021). N-Acetylcysteine and Hydrogen Sulfide in Coronavirus Disease 2019. Antioxid. Redox Signal..

[10] Agrawal R., Pal V.K., Suhas K.S., Menon G.J., Singh I.R., Malhotra N., Naren C.S., Ganesh K., Rajmani R.S., Seshasayee A.S.N. (2025). Hydrogen Sulfide (H2S) Coordinates Redox Balance, Carbon Metabolism, and Mitochondrial Bioenergetics to Suppress SARS-CoV-2 Infection. PLoS Pathog..

[11] Datta P.K., Liu F., Fischer T., Rappaport J., Qin X. (2020). SARS-CoV-2 Pandemic and Research Gaps: Understanding SARS-CoV-2 Interaction with the ACE2 Receptor and Implications for Therapy. Theranostics.

[12] Gheblawi M., Wang K., Viveiros A., Nguyen Q., Zhong J.-C., Turner A.J., Raizada M.K., Grant M.B., Oudit G.Y. (2020). Angiotensin-Converting Enzyme 2: SARS-CoV-2 Receptor and Regulator of the Renin-Angiotensin System. Circ. Res..

[13] Beyerstedt S., Casaro E.B., Rangel É.B. (2021). COVID-19: Angiotensin-Converting Enzyme 2 (ACE2) Expression and Tissue Susceptibility to SARS-CoV-2 Infection. Eur. J. Clin. Microbiol. Infect. Dis..

[14] Ajoolabady A., Pratico D., Ren J. (2024). Angiotensin II: Role in Oxidative Stress, Endothelial Dysfunction, and Diseases. Mol. Cell. Endocrinol..

[15] Bhullar S.K., Dhalla N.S. (2022). Angiotensin II-Induced Signal Transduction Mechanisms for Cardiac Hypertrophy. Cells.

[16] Lin Y., Zeng H., Gao L., Gu T., Wang C., Zhang H. (2017). Hydrogen Sulfide Attenuates Atherosclerosis in a Partially Ligated Carotid Artery Mouse Model via Regulating Angiotensin Converting Enzyme 2 Expression. Front. Physiol..

[17] Shang J., Wan Y., Luo C., Ye G., Geng Q., Auerbach A., Li F. (2020). Cell Entry Mechanisms of SARS-CoV-2. Proc. Natl. Acad. Sci. USA.

[18] Yang G. (2020). H2S as a Potential Defense against COVID-19?. Am. J. Physiol.-Cell Physiol..

[19] Bazhanov N., Escaffre O., Freiberg A.N., Garofalo R.P., Casola A. (2017). Broad-Range Antiviral Activity of Hydrogen Sulfide Against Highly Pathogenic RNA Viruses. Sci. Rep..

[20] Pal V.K., Bandyopadhyay P., Singh A. (2018). Hydrogen Sulfide in Physiology and Pathogenesis of Bacteria and Viruses. IUBMB Life.

[21] Li H., Ma Y., Escaffre O., Ivanciuc T., Komaravelli N., Kelley J.P., Coletta C., Szabo C., Rockx B., Garofalo R.P. (2015). Role of Hydrogen Sulfide in Paramyxovirus Infections. J. Virol..

[22] Bazhanov N., Ivanciuc T., Wu H., Garofalo M., Kang J., Xian M., Casola A. (2018). Thiol-Activated Hydrogen Sulfide Donors Antiviral and Anti-Inflammatory Activity in Respiratory Syncytial Virus Infection. Viruses.

[23] Catanzaro M., Fagiani F., Racchi M., Corsini E., Govoni S., Lanni C. (2020). Immune Response in COVID-19: Addressing a Pharmacological Challenge by Targeting Pathways Triggered by SARS-CoV-2. Signal Transduct. Target. Ther..

[24] Van Tin H., Rethi L., Higa S., Kao Y.-H., Chen Y.-J. (2024). Spike Protein of SARS-CoV-2 Activates Cardiac Fibrogenesis through NLRP3 Inflammasomes and NF-κB Signaling. Cells.

[25] Zhang D., Wang X., Chen S., Chen S., Yu W., Liu X., Yang G., Tao Y., Tang X., Bu D. (2019). Endogenous Hydrogen Sulfide Sulfhydrates IKKβ at Cysteine 179 to Control Pulmonary Artery Endothelial Cell Inflammation. Clin. Sci..

[26] Zhao H., Liu H., Yang Y., Wang H. (2022). The Role of H2S Regulating NLRP3 Inflammasome in Diabetes. Int. J. Mol. Sci..

[27] Xia Y., Zhang W., He K., Bai L., Miao Y., Liu B., Zhang X., Jin S., Wu Y. (2023). Hydrogen Sulfide Alleviates Lipopolysaccharide-Induced Myocardial Injury through TLR4-NLRP3 Pathway. Physiol. Res..

[28] Huang D., Jing G., Zhu S. (2023). Regulation of Mitochondrial Respiration by Hydrogen Sulfide. Antioxid. Basel Switz..

[29] Paul B.D., Snyder S.H., Kashfi K. (2021). Effects of Hydrogen Sulfide on Mitochondrial Function and Cellular Bioenergetics. Redox Biol..

[30] Módis K., Asimakopoulou A., Coletta C., Papapetropoulos A., Szabo C. (2013). Oxidative Stress Suppresses the Cellular Bioenergetic Effect of the 3-Mercaptopyruvate Sulfurtransferase/Hydrogen Sulfide Pathway. Biochem. Biophys. Res. Commun..

[31] Sbodio J.I., Snyder S.H., Paul B.D. (2018). Golgi Stress Response Reprograms Cysteine Metabolism to Confer Cytoprotection in Huntington’s Disease. Proc. Natl. Acad. Sci. USA.

[32] Zhang Y., Wang Y., Read E., Fu M., Pei Y., Wu L., Wang R., Yang G. (2020). Golgi Stress Response, Hydrogen Sulfide Metabolism, and Intracellular Calcium Homeostasis. Antioxid. Redox Signal..

[33] Kabil O., Yadav V., Banerjee R. (2016). Heme-Dependent Metabolite Switching Regulates H2S Synthesis in Response to Endoplasmic Reticulum (ER) Stress. J. Biol. Chem..

[34] Hine C., Harputlugil E., Zhang Y., Ruckenstuhl C., Lee B.C., Brace L., Longchamp A., Treviño-Villarreal J.H., Mejia P., Ozaki C.K. (2015). Endogenous Hydrogen Sulfide Production Is Essential for Dietary Restriction Benefits. Cell.

[35] Kandil S., Brennan L., McBean G.J. (2010). Glutathione Depletion Causes a JNK and p38MAPK-Mediated Increase in Expression of Cystathionine-γ-Lyase and Upregulation of the Transsulfuration Pathway in C6 Glioma Cells. Neurochem. Int..

[36] Martín J.A., Pereda J., Martínez-López I., Escrig R., Miralles V., Pallardó F.V., Viña J.R., Vento M., Viña J., Sastre J. (2007). Oxidative Stress as a Signal to Up-Regulate Gamma-Cystathionase in the Fetal-to-Neonatal Transition in Rats. Cell. Mol. Biol..

[37] Ibrahim H., Perl A., Smith D., Lewis T., Kon Z., Goldenberg R., Yarta K., Staniloae C., Williams M. (2020). Therapeutic Blockade of Inflammation in Severe COVID-19 Infection with Intravenous N-Acetylcysteine. Clin. Immunol..

[38] Zhou N., Yang X., Huang A., Chen Z. (2021). The Potential Mechanism of N-Acetylcysteine in Treating COVID-19. Curr. Pharm. Biotechnol..

[39] Milara J., Martínez-Expósito F., Montero P., Roger I., Bayarri M.A., Ribera P., Oishi-Konari M.N., Alba-García J.R., Zapater E., Cortijo J. (2022). N-Acetylcysteine Reduces Inflammasome Activation Induced by SARS-CoV-2 Proteins In Vitro. Int. J. Mol. Sci..

[40] Ezeriņa D., Takano Y., Hanaoka K., Urano Y., Dick T.P. (2018). N-Acetyl Cysteine Functions as a Fast-Acting Antioxidant by Triggering Intracellular H2S and Sulfane Sulfur Production. Cell Chem. Biol..

[41] Renieris G., Katrini K., Damoulari C., Akinosoglou K., Psarrakis C., Kyriakopoulou M., Dimopoulos G., Lada M., Koufargyris P., Giamarellos-Bourboulis E.J. (2020). Serum Hydrogen Sulfide and Outcome Association in Pneumonia by the SARS-CoV-2 Coronavirus. Shock.

[42] Varikasuvu S.R., Manne M., Kumar S., Mudgal S.K., Raj V., Varshney S., Gupta P., Grover A., Goyal C., Lal V. (2025). COVID-19 Clinical Outcomes and N-Acetylcysteine (CoViNAC Study): A GRADE Compliant Meta-Analysis of Randomized Controlled Trials with Molecular Docking and Dynamics Simulation Studies with Mpro of SARS-CoV-2. Cell. Mol. Biol..

[43] Fratta Pasini A.M., Stranieri C., Di Leo E.G., Bertolone L., Aparo A., Busti F., Castagna A., Vianello A., Chesini F., Friso S. (2025). Identification of Early Biomarkers of Mortality in COVID-19 Hospitalized Patients: A LASSO-Based Cox and Logistic Approach. Viruses.

[44] Shen X., Pattillo C.B., Pardue S., Bir S.C., Wang R., Kevil C.G. (2011). Measurement of Plasma Hydrogen Sulfide in Vivo and in Vitro. Free Radic. Biol. Med..

[45] Park E.K., Jung H.S., Yang H.I., Yoo M.C., Kim C., Kim K.S. (2007). Optimized THP-1 Differentiation Is Required for the Detection of Responses to Weak Stimuli. Inflamm. Res. Off. J. Eur. Histamine Res. Soc. Al.

[46] Peserico D., Stranieri C., Garbin U., Mozzini C.C., Danese E., Cominacini L., Fratta Pasini A.M. (2020). Ezetimibe Prevents Ischemia/Reperfusion-Induced Oxidative Stress and Up-Regulates Nrf2/ARE and UPR Signaling Pathways. Antioxidants.

[47] Fratta Pasini A., Anselmi M., Garbin U., Franchi E., Stranieri C., Nava M.C., Boccioletti V., Vassanelli C., Cominacini L. (2007). Enhanced Levels of Oxidized Low-Density Lipoprotein Prime Monocytes to Cytokine Overproduction via Upregulation of CD14 and Toll-like Receptor 4 in Unstable Angina. Arterioscler. Thromb. Vasc. Biol..

[48] Johnson S., Nguyen V., Coder D. (2013). Assessment of Cell Viability. Curr. Protoc. Cytom..

[49] Adan A., Kiraz Y., Baran Y. (2016). Cell Proliferation and Cytotoxicity Assays. Curr. Pharm. Biotechnol..

[50] Khalef L., Lydia R., Filicia K., Moussa B. (2024). Cell Viability and Cytotoxicity Assays: Biochemical Elements and Cellular Compartments. Cell Biochem. Funct..

[51] Vermes I., Haanen C., Steffens-Nakken H., Reutelingsperger C. (1995). A Novel Assay for Apoptosis. Flow Cytometric Detection of Phosphatidylserine Expression on Early Apoptotic Cells Using Fluorescein Labelled Annexin V. J. Immunol. Methods.

[52] Van Engeland M., Ramaekers F.C.S., Schutte B., Reutelingsperger C.P.M. (1996). A Novel Assay to Measure Loss of Plasma Membrane Asymmetry during Apoptosis of Adherent Cells in Culture. Cytometry.

[53] Di Leo E.G., Stranieri C., Zoccatelli G., Bellumori M., Zonfrillo B., Cominacini L., Fratta Pasini A.M. (2025). Olive Pomace Extract Acts as a New Potent Ferroptosis Inhibitor in Human Cells. Molecules.

[54] Celeghini E.C.C., Alves M.B.R., De Arruda R.P., De Rezende G.M., Florez-Rodriguez S.A., De Sá Filho M.F. (2021). Efficiency of CellROX Deep Red ^®^ and CellROX Orange ^®^ Fluorescent Probes in Identifying Reactive Oxygen Species in Sperm Samples from High and Low Fertility Bulls. Anim. Biotechnol..

[55] Drummen G.P.C., van Liebergen L.C.M., Op den Kamp J.A.F., Post J.A. (2002). C11-BODIPY(581/591), an Oxidation-Sensitive Fluorescent Lipid Peroxidation Probe: (Micro)Spectroscopic Characterization and Validation of Methodology. Free Radic. Biol. Med..

[56] Pap E.H.W., Drummen G.P.C., Winter V.J., Kooij T.W.A., Rijken P., Wirtz K.W.A., Op den Kamp J.A.F., Hage W.J., Post J.A. (1999). Ratio-Fluorescence Microscopy of Lipid Oxidation in Living Cells Using C11-BODIPY581/591. FEBS Lett..

[57] Enomoto A.C., Schneider E., McKinnon T., Goldfine H., Levy M.A. (2020). Validation of a Simplified Procedure for Convenient and Rapid Quantification of Reduced and Oxidized Glutathione in Human Plasma by Liquid Chromatography Tandem Mass Spectrometry Analysis. Biomed. Chromatogr. BMC.

[58] Razali N.M., Wah Y.B. (2011). Power Comparisons of Shapiro-Wilk, Kolmogorov-Smirnov, Lilliefors and Anderson-Darling Tests. J. Stat. Model. Anal..

[59] Wang M., Guo Z., Wang S. (2014). Regulation of Cystathionine γ-Lyase in Mammalian Cells by Hypoxia. Biochem. Genet..

[60] Wang M., Guo Z., Wang S. (2014). The Binding Site for the Transcription Factor, NF-κB, on the Cystathionine γ-Lyase Promoter Is Critical for LPS-induced Cystathionine γ-Lyase Expression. Int. J. Mol. Med..

[61] Sen N., Paul B.D., Gadalla M.M., Mustafa A.K., Sen T., Xu R., Kim S., Snyder S.H. (2012). Hydrogen Sulfide-Linked Sulfhydration of NF-κB Mediates Its Antiapoptotic Actions. Mol. Cell.

