# Supplementary material for: Decreased Plasma Concentration of Hydrogen Sulfide in Hospitalized COVID-19 Patients: A Novel Determinant of Mortality?
Source: Antioxidants (Basel). 2026 Feb 28;15(3):307. doi: 10.3390/antiox15030307 (PMC13023920; doi:10.3390/antiox15030307)
Supplement: Supplementary file 1 [file antioxidants-15-00307-s001.zip › antioxidants-4024027-supplementary.pdf]

TABLE S1. ANTHROPOMETRIC, CLINICAL CHARACTERISTICS AND ROUTINE LABORATORY PARAMETERS IN THE THREE GROUPS OF SUBJECTS PARTICIPATING IN THE STUDY

	Non-COVID subjects (n. 40)	Survivor COVID patients (n. 40)	Non-survivor COVID patients (n. 40)	p-value
**Age**	75.0 [68.5-77.8]	75.5 [73-84]	80.5 [76.2-86]	NS
**Sex (M/F)**	20/20	21/19	21/19	NS
**Comorbidities**				
**Hypertension n. (%)**	16 (40)	24 (60)	18 (45)	NS
**Diabetes mellitus n. (%)**	3 (7.5)	9 (22.5) **	4 (10)	<0.01
**Chronic kidney disease n. (%)**	0	3 (7.5)	5 (12.5)	NS
**Chronic liver disease n. (%)**	0	2	2	NS
**PaO2/FiO2** (ratio)	NA	281.0 [229.0-314.0]	224.0 [162.0-252.0]	<0.0001
**Admission to ICU** n. (%)	NA	4 (10)	8 (20)	<0.001
**Days of hospitalization** n. (%)	NA	15.0 [8.0-21.0]	11.5 [8.0-17.3]	NS
**In-hospital death n. (%)**	NA	0	40 (100)	<0.0001
**Biochemical parameters**				
**Creatinine** (mg/dL)	0.8 [0.7-1.0]	0.8 [0.7-1.0]	1.0 [0.7-1.5]	NS
**AST** (U/L)	23.5 [21.0-27.0]	35.0 [26.0-52.0] *	38.5 [30.0-47.0] *	<0.001
**ALT** (U/L)	21.5 [17.0-27.0)	25.0 [17-5-47.5]	27.0 [21.0-37.3]	NS
**CRP (mg/L)**	1 [0.07-4.0)	68 [36-127] *	78 [44-150.5] *	<0.0001

Legend: Data are presented as n (%) or median [interquartile range]. Statistical significance was set at p<0.05; ALT: alanine aminotransferase; AST: aspartate aminotransferase; ICU: intensive care unit; PaO2: arterial partial oxygen tension; FiO2: inspiratory oxygen fraction. * p< vs non- COVID; ** p< vs non-COVID and non-survivors.

TABLE S2: IRON METABOLISM PARAMETERS AND COMPLETE BLOOD COUNT IN THE THREE GROUPS OF SUBJECTS PARTICIPATING IN THE STUDY

	**Non-COVID subjects (n. 40)**	**Survivor COVID patients (n. 40)**	**Non-survivor COVID patients (n. 40)**	**p-value**
**Iron** (mcg/dl)	91.0 [79-117]	33.9 [24.4-53.8] *	35.1 [25.5-49.8] *	<0.0001
**Transferrin** (g/L)	2.5 [2.4-2.8]	1.5 [1.3-1.9] *	1.6 [1.1-1.9] *	<0.0001
**Transferrin sat.** (%)	26.5 [21-32.2]	16 [11-26] *	16 [11-27] *	<0.01
**Ferritin** (mcg/L)	151 [24-201]	870 [82-1265] *	813.5 [504-1574] *	<0.0001
**Hematocrit** (%)	43.5 [41.2-45]	40.5 [41.2-45]	39.0 [34-43]	NS
**Hb** (g/dl)	14.5 [13.4-15.3]	13.1 [11.1-13.8] *	12.5 [12-14] *	<0.001
**RBC** (10^{12}/L)	4.8 [4.4-5.1]	4.3 [3.8-4.8] *	4.2 [3.8-4.7] *	<0.001
**RDW** (%)	13.1 [12.6-13.8]	13.5 [12.6-13.8]	14.3 [12.7-14.9] **	<0.01
**Platelets** (10^9/L)	225.5 [189-282.5]	212 [177.3-271.3]	188 [132-234] *	<0.05
**WBC** (10^9/L)	5.8 [5.1-6.6]	6.8 [5.2-9.9]	6.5 [4.3-10.9]	NS
**Neutrophils** (10^9/L)	3.3 [2.9-4.0]	5.2 [4-7.7] ***	5.3 [3.1-9.2] ***	<0.001
**Lymphocytes** (10^9/L)	1.6 [1.3-2.1]	0.7 [0.5-1.1] ****	0.5 [0.4-0.9] ****	<0.0001
**Monocytes** (10^9/L)	0.5 [0.4-0.56]	0.34 [0.3-0.7]	0.24 [0.1-0.4] ***; §	<0.001
**Basophils** (10^9/L)	0.03 [0.02-0.04]	0 [0-0.01] ****	0 [0-0.01] ****	<0.0001

Legend. Data are presented as median [interquartile range]. Statistical significance was set at p<0.05. RBC: red blood cells, RDW: red cell distribution width, WBC: white blood cells. * p< vs controls; ** p < 0.01, *** p < 0.001 and **** p < 0.0001; §p <0.05 vs survivors.

TABLE S3: EFFECT SIZES WITH COHEN'S d METHOD FOR H_2S AND MDA

Variabile	Cohen's d	IC 95% inf	IC 95% sup	Magnitude
H_2S	0.744	0.284	1.204	medium
MDA	-0.688	-1.146	-0.230	medium

FIGURE S1: CORRELATION HEATMAP

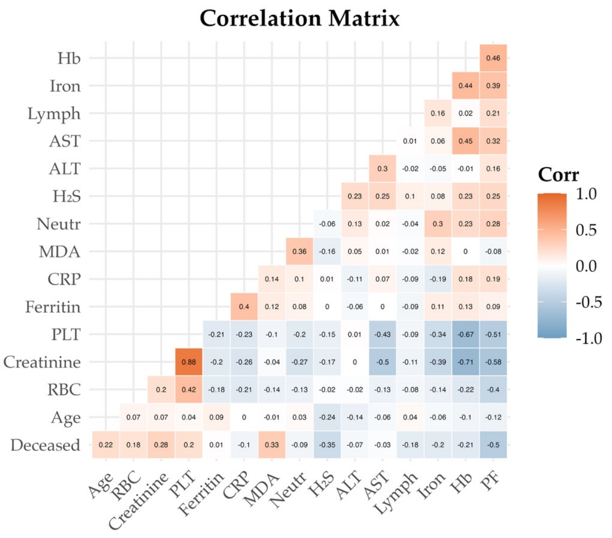

Figure S2: SURVIVAL CURVES IN COVID-19 PATIENTS

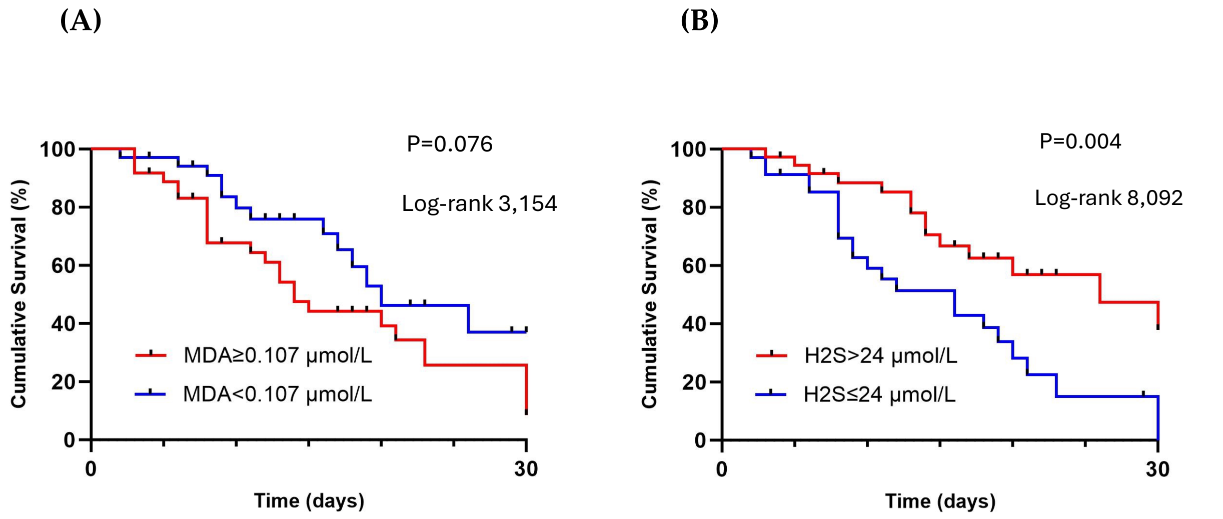

Kaplan-Meier curves showing survival at 30 days in COVID-19 patients in relation to median plasma concentrations of MDA (A) and H_2S (B).

Figure S3: ROC CURVE ANALYSIS FOR H_2S AS MORTALITY PREDICTOR

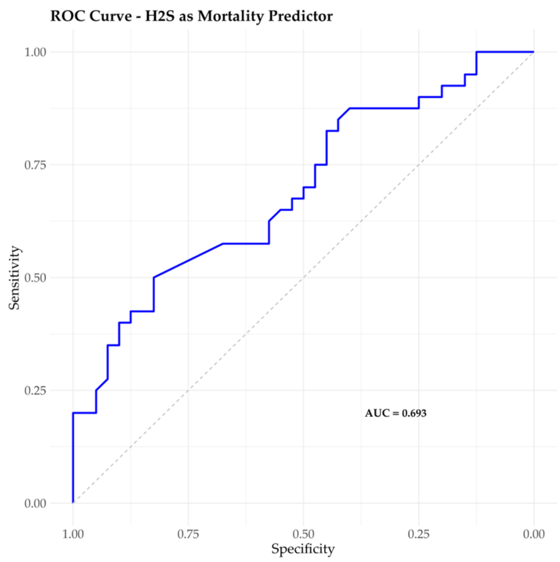

Parameter	H_2S
AUC	0.693
95% CI	0.578 - 0.809
Optimal Cutoff	20.65
Sensitivity	50%
Specificity	82.5%
PPV	74.1%
NPV	62.3%
Accuracy	66.2%
